# AIstain: Enhancing microglial phagocytosis analysis through deep learning

**DOI:** 10.1016/j.crmeth.2025.101207

**Published:** 2025-10-17

**Authors:** Alexander Zähringer, Janaki Manoja Vinnakota, Tobias Wertheimer, Philipp Saalfrank, Marie Follo, Florian Ingelfinger, Robert Zeiser

**Affiliations:** 1 Department of Medicine I, Medical Center, University of Freiburg, Faculty of Medicine, Albert-Ludwigs-University Freiburg, Freiburg im Breisgau, Germany; 2 Lighthouse Core Facility, Faculty of Medicine, Albert-Ludwigs-University Freiburg, Freiburg im Breisgau, Germany; 3 Signaling Research Centers BIOSS and CIBSS, Center for Integrative Biological Signaling Studies, Albert-Ludwigs-University Freiburg, Freiburg im Breisgau, Germany

**Keywords:** microglia, phagocytosis, neuroinflammation, neural network, U-Net, artificial intelligence

## Abstract

Investigating microglial phagocytosis is essential for understanding the mechanisms underlying brain health and disease. Dysregulation of phagocytosis is implicated in various neurological disorders, necessitating accurate analysis. Leveraging advances in deep learning, this study explores the application of a U-Net-based neural network for image cytometry to enhance the analysis of microglial phagocytosis. Murine microglia were imaged using the Olympus ScanR system, generating a substantial dataset for training a U-Net. The U-Net (AIstain) demonstrated superior performance in cell detection compared to live cell staining and the established segmentation tools SAM2 and Cellpose 3. Additionally, the model’s applicability can be extended to other cell types, including leukemia and breast cancer cells, highlighting its versatility. AIstain provides a straightforward approach for the analysis of live cell images and microglial phagocytosis. This method enhances the precision of the results while simultaneously reducing the complexity of the experiment, thus facilitating substantial progress in the domain of neurobiological research.

## Highlights

• AIstain enables label-free microglial detection in live cell image cytometry
• AIstain demonstrates superior performance compared to live cell staining
• Publicly available and easy-to-use experimental setup for phagocytosis assays
• Broad applicability on other cell types beyond microglia

## Motivation

Understanding microglial phagocytosis is a key to developing therapies for neuroinflammatory diseases. Current methods for live cell phagocytosis assays involve cell labeling, which can cause toxicity and alter cell function. To address this, we developed a deep learning-based approach that avoids using additional reagents, ensuring a more physiological environment for studying microglia in live cell imaging. Our neural network can be easily implemented in Olympus ScanR systems and has also demonstrated broader applications beyond microglial phagocytosis.

## Graphical abstract

**Figure:**
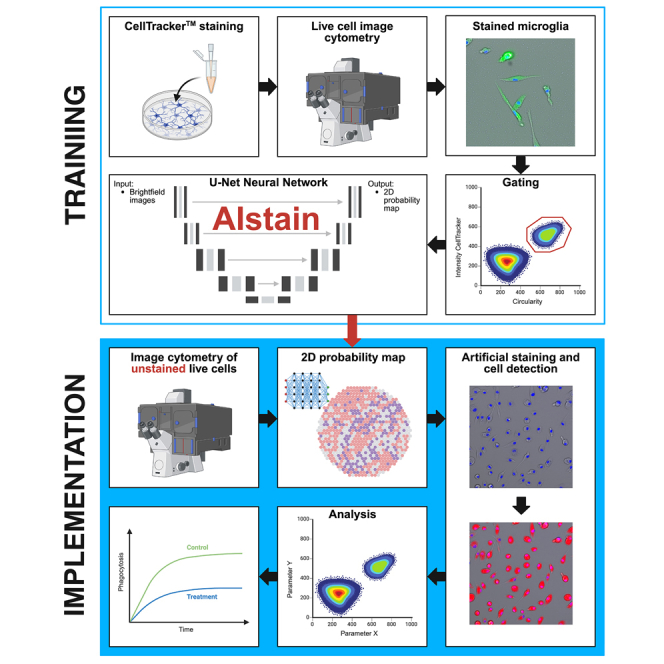

## Teaser

Zähringer et al. introduce AIstain, a U-Net-based deep learning approach for label-free microglial detection in live cell image cytometry. Their method outperforms fluorescence staining and common segmentation tools, enabling accurate, high-throughput phagocytosis analysis while avoiding cytotoxic dyes.

## Introduction

Microglia, the resident immune cells of the brain, play a pivotal role in maintaining brain homeostasis not only by clearing debris and pathogens through phagocytosis but also by mediating brain inflammation.^1^^,^^2^^,^^3^^,^^4^ Dysregulation of this process is implicated in various neurological disorders, including Alzheimer’s disease, multiple sclerosis, and other neuroinflammatory diseases^1^ like graft-versus-host disease of the central nervous system.^4^ Therefore, accurate and efficient analysis of microglial phagocytosis is essential for advancing neurobiological research, therapeutic development, and the understanding of the cellular and molecular mechanisms of underlying brain health and disease.^5^ Current methods to study phagocytosis employ flow cytometry, which does not allow cell tracking over time, or live cell imaging or high content analysis (HCA).^6^^,^^7^ For live cell imaging, the cells need to be stained using fluorescent dyes^8^ to allow cell detection by subsequent image analysis. However, it was shown that cell labeling affects the phagocytic behavior of macrophages, leading to inaccurate results^9^ and pointing out the need for other methods without cell labeling.

Recent advances in deep learning, particularly convolutional neural networks (CNNs), have significantly enhanced the capabilities of fluorescence microscopy and HCA/image cytometry.^10^^,^^11^^,^^12^ Deep learning algorithms, with their ability to learn and generalize from large datasets,^13^^,^^14^ have revolutionized the field of image analysis, offering improved efficiency in processing complex biological images.^15^ In HCA, deep learning techniques have been successfully employed for tasks such as cell segmentation.^15^^,^^16^ In addition to segmentation, deep learning models have demonstrated remarkable performance in the detection and classification of cells.^17^ CNNs can classify cell types, assess cell viability, and identify specific cellular behaviors by learning from morphological and fluorescence patterns present in microscopy images.^18^^,^^19^^,^^20^ This automated analysis is crucial for high-throughput studies, enabling the rapid processing of large datasets and the extraction of meaningful biological insights.^17^ *In silico* staining, powered by CNNs, can generate predictions of fluorescent labels from unstained cells,^21^^,^^22^ thereby reducing the need for potentially disruptive exogenous labeling.^9^^,^^23^^,^^24^^,^^25^ This approach is especially valuable in live cell imaging, where maintaining cell viability is crucial.^23^ U-Nets, with their distinctive encoder-decoder structure, can capture both global context and fine details,^26^ making them particularly effective for *in silico* staining and segmenting individual cells and subcellular structures from microscopy images.^10^^,^^15^^,^^26^

Overall, the integration of deep learning into HCA of living cells enables automated, accurate, and high-throughput analysis of complex microscopy datasets enhancing our understanding of cellular processes.^27^^,^^28^ This study explores the application of the publicly available deep learning model AIstain for improved accuracy and a more physiological environment in HCA to better understand the process of microglial phagocytosis and its role in brain’s health and disease.

## Results

### Neural network training

To establish a training and validation dataset for a U-Net-based deep learning approach, called AIstain, which can be used for live cell imaging of microglial phagocytosis using the Olympus ScanR system, we stained living (unfixed) primary murine microglia, with CellTracker orange (cytoplasm stain) and Hoechst 33342 (nuclear stain) prior to imaging. pHrodo Deep Red phagocytosis beads (Invitrogen) were added according to the manufacturer’s instructions before acquiring the training/validation dataset images to mimic the experimental settings of phagocytosis assays. This allowed us to increase the background noise over time in the training dataset as the beads sediment onto the bottom of the plate. The microglia were imaged continuously once every 15 min for a period of 3.5 h using the Olympus ScanR high content system. Thereby, we generated a training dataset (2,240 images) and a validation dataset (560 images) (split 80%). Using the Olympus ScanR analysis software, microglia were selected and gated using CellTracker mean fluorescence intensity (MFI) and Hoechst 33342 MFI nuclear signal to only include viable cells and exclude cell debris in the training process as described in STAR Methods and shown in Figures S1A and S1B. To enhance accuracy, stringent gating was applied to enable that only clear CellTracker staining was used as the basis for training. The gated microglia, thereby labeled as a cell, were used to train a deep neural network with U-Net architecture. The detailed settings and architecture used are described in STAR Methods. The U-Net used the CellTracker fluorescence staining paired with the bright-field image of the same cell to recognize cells in bright-field images. The correct alignment of the fluorescence and bright-field signal was ensured by using images that each consist of a fluorescence and a bright-field mask. In this way, 25,000 iterations on the training dataset were done. The training performance was monitored using the pixel-wise Jaccard fuzzy index (similarity) (Figure S1C).

Performing a 5-fold cross-validation demonstrated similar training dynamics across each cross-validation split monitored by the Jaccard fuzzy index (Figure S2A) compared to the AIstain (full training dataset). Furthermore, we could show that each of the cross-validation splits showed similar area under the curve of the receiver operator characteristic curve (AUC ROC), sensitivity, and F1 scores (Figure S2B), indicating the robustness of AIstain.

AIstain was implemented as a new virtual channel on the microscope. Further details on how to implement AIstain in the Olympus ScanR software are described in STAR Methods. The virtual channel creates a 2D probability map, resembling an artificial *in silico* staining. This artificial staining can be used in the same way as fluorescence cell staining to segment cells and gate for cells in the imaging analysis (Figure S3A).

### AIstain shows better performance than classic staining or segmentation tools Cellpose 3 and SAM2

**Figure 1:**
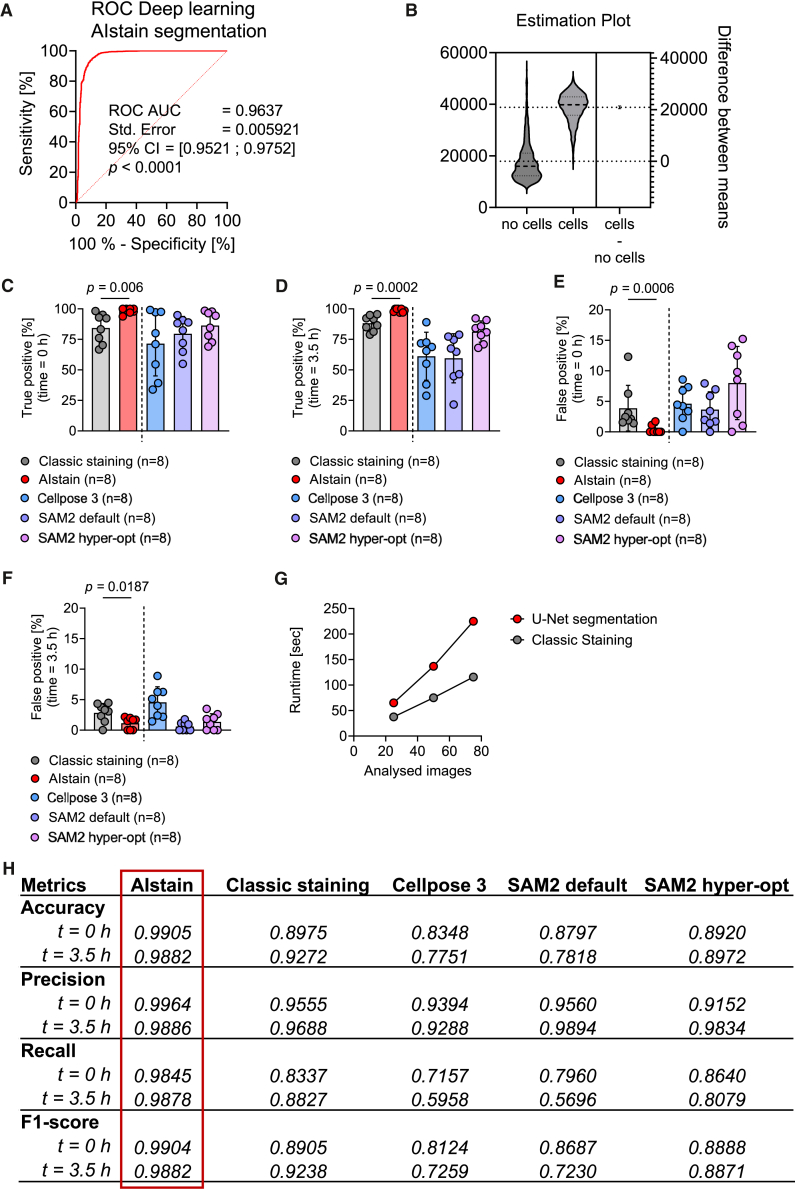
Improved performance of U-Net-based segmentation compared to classic staining: Cellpose 3 and SAM2 (A) Receiver operator curve showing neural network performance on primary microglia. Dataset contains 2.884 entries; 95% confidence interval was chosen with Wilson/Brown method. (B) Estimation plot showing the distribution of the neural network prediction value (16-bit scale, the values 0, 32,767, and 65,535 correspond to classification probabilities of 0%, 50%, and 100%) for the classes no cells and cells. The neural network prediction value resembles the probability of the recognized object being a cell. The difference between means shows the difference of the mean prediction value in the class no cells compared to difference of the mean prediction value in the class cells. The higher this value is, the better the segregation of the two classes. (C–F) Scatter dot plots showing percentage of (C) true-positive detection at time point 0 h of classic staining (*n* = 8), AIstain (*n* = 8), Cellpose 3 (*n* = 8), SAM2 default (*n* = 8), and SAM2 hyperopt settings (*n* = 8); (D) true-positive detection at time point 3.5 h of classic staining (*n* = 8), AIstain (*n* = 8), Cellpose 3 (*n* = 8), SAM2 default (*n* = 8), and SAM2 hyperopt settings (*n* = 8); (E) false-positive detection at time point 0 h of classic staining (*n* = 8), AIstain (*n* = 8), Cellpose 3 (*n* = 8), SAM2 default (*n* = 8), and SAM2 hyperopt settings (*n* = 8); and (F) false-positive detection at time point 3.5 h of classic staining (*n* = 8), AIstain (*n* = 8), Cellpose 3 (*n* = 8), SAM2 default (*n* = 8), and SAM2 hyperopt settings (*n* = 8). *p* values calculated using (C and E) unpaired t test or (C, F) Mann-Whitney test. Error bars indicating mean ± SEM. (G) Plot showing analysis time in seconds depending on number of analyzed images. (H) Table showing performance metrics of U-Net segmentation compared to classic staining Cellpose 3, SAM2 default, and SAM2 hyperopt settings.

To evaluate the performance of AIstain and to compare it to the classic staining we used the validation dataset (20%). AIstain showed a good overall detection performance with an AUC ROC) of 0.9637 (Figure 1A). Moreover, the detection probabilities segregate well between the truth (cell) and false (no cell) (Figure 1B). The ground truth was determined by manual annotation combined with re-gating of the biological parameters of the detection such as area and circularity. Comparison with the live cell staining using CellTracker revealed significantly improved true-positive rates at t = 0 h and even at t = 3.5 h with increased background noise due to the phagocytosis particles (Figures 1C–1F). We selected time point t = 0 h and t = 3.5 h as they represent the two extremes of background noise of the images. At t = 0 h, there is almost no background noise because the phagocytosis beads are still in suspension in the culture medium (image acquired immediately after adding the phagocytosis beads to the cells). Over time, these beads start to sediment onto the bottom of the plate, resulting in increasing background noise.

Moreover, the widely used segmentation algorithm Cellpose 3,^29^ which is based on a U-Net architecture, showed lower true-positive rates for detecting microglia. Also, the advanced segmentation approach SAM2, a segmentation tool using the Hiera architecture,^30^^,^^31^ showed worse performance than AIstain, using both the default settings and hyperopt settings (Figures 1C–1F and S4A–S4O). Along with that, the false-positive rate was reduced using our deep learning-based U-Net segmentation AIstain compared to CellTracker, Cellpose 3, and SAM2 (Figures 1E and 1F). It should be emphasized that, even with increased background in the bright-field image at t = 3.5 h due to the sedimented phagocytosis beads, false-positive rates are lower than using classic staining. Moreover, the established segmentation tools Cellpose 3 and SAM2 showed higher false-positive rates at t = 0 h (Figures 1C–1F and S4A–S4O). The analysis runtime of the computing increased compared to the analysis of a classic cell staining (Figure 1G).

**Figure 2:**
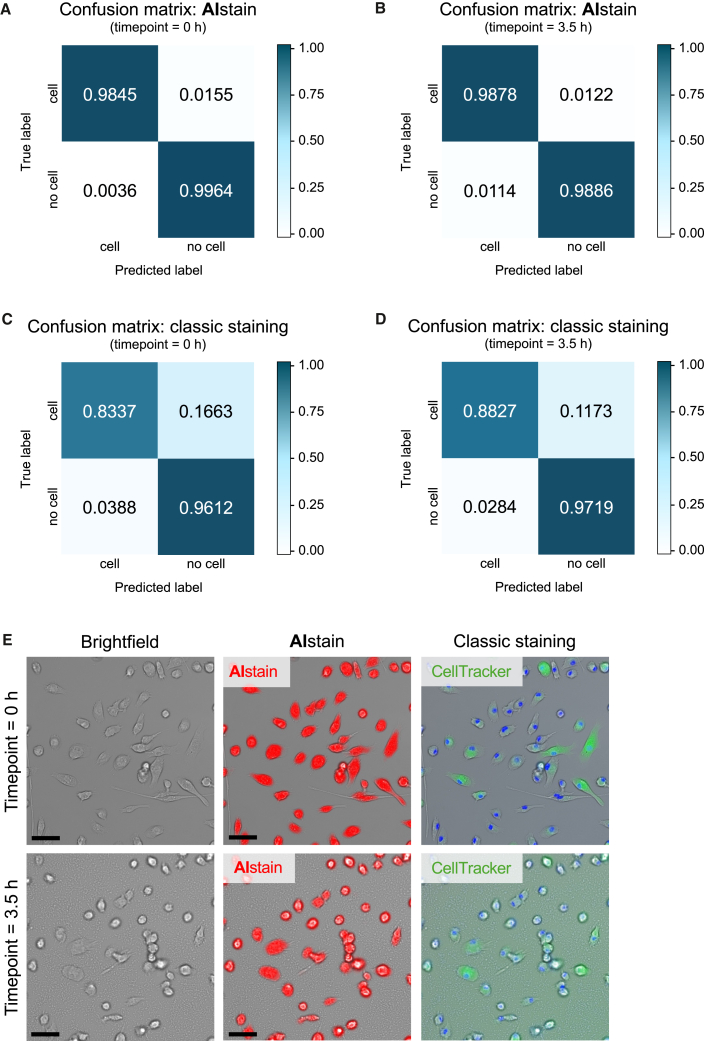
U-Net-based segmentation demonstrates higher detection rates than classic staining (A–D) Confusion matrices showing performance of cell labeling of (A) U-Net segmentation at time point 0 h, (B) U-Net segmentation at time point 3.5 h, (C) classic staining at time point 0 h, and (D) classic staining at time point 3.5 h. Values calculated from predicted vs. manual annotations. (E) Representative images showing deep learning artificial *in silico* staining (red) compared to classic staining (green) of primary microglia at time point 0 h (upper panel) and time point 3.5 h (lower panel). Scale bars: 50 μm.

The summarized performance metrics of AIstain, classic staining, Cellpose 3, and SAM2 underscore the high accuracy and performance of AIstain on unseen images (Figure 1H). A summary of the overall confusion of AIstain and classic staining demonstrates the superior performance of the deep learning model compared to classic cell staining (Figures 2A–2D), Cellpose 3 and SAM2 (Figures S4M–S4O). The high accuracy of cell detection by AIstain is also shown in the representative images in Figure 2E. In summary, the performance of AIstain in detecting unlabeled microglia with their different morphology excelled live cell staining and the commonly used segmentation tools Cellpose 3 and SAM2.

### AIstain improves the workflow of live cell high content analysis of microglial phagocytosis

Our initial purpose was to train a neural network that can be used to facilitate phagocytosis assays in live cell HCA using the Olympus ScanR system and to avoid the use of toxic dyes. Commonly used phagocytosis assays need cell labeling in order to achieve single-cell resolution of phagocytosis.^8^ However, live cell dyes are toxic to the cells,^25^ especially phototoxicity plays a relevant role if the cells are imaged for multiple hours. We now describe a label-free approach with single-cell resolution in phagocytosis analysis without the need of toxic and time-consuming cell staining procedures.

**Figure 3:**
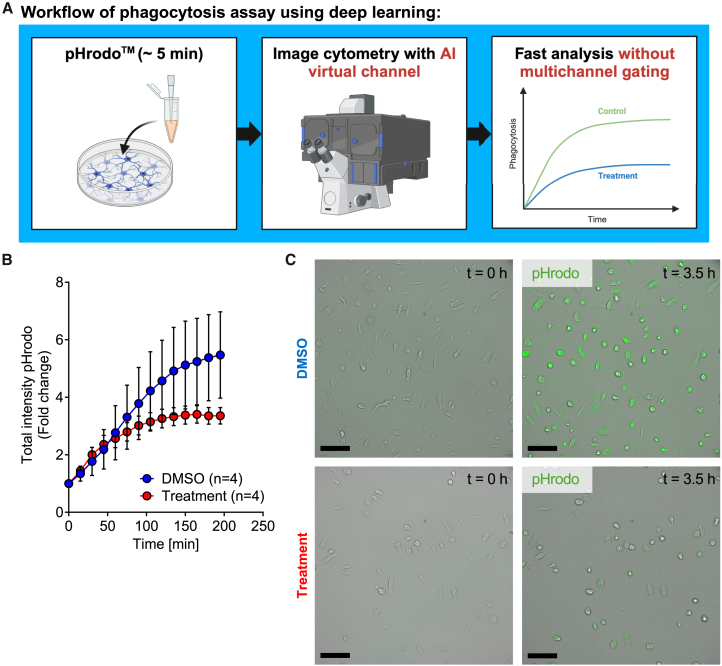
U-Net-based segmentation improves the workflow of live cell high content analysis of microglial phagocytosis (A) Graphical overview of a workflow for the implementation of deep learning in a phagocytosis assay. (B) Line graph showing time-dependent pHrodo uptake of primary microglia treated with either DMSO (*n* = 4) or 6-formylindolo(3,2-b)carbazole (FICZ) (*n* = 4). Each single *n* resembles an independent biological replicate (independent primary microglia culture derived from two mice per culture). Approximately 5,000 cells from each individual culture were analyzed per data point and time point. Analysis done using the neural network for cell detection. Error bars indicating mean ± SEM. *p* value calculated using nonlinear fit. (C) Representative images showing pHrodo uptake of primary microglia treated with either DMSO or FICZ. Scale bars: 100 μm.

Microglia, or the cells of interest, are grown and treated directly in a 96-well glass-bottom plate. Prior to imaging, there is only the need to exchange the culture medium with live cell imaging solution and to add the phagocytosis beads. This process is relatively simple and needs no longer than 5 min (depending on the number of experimental conditions). Afterward, the cells can be directly mounted on the Olympus ScanR live cell imaging system (Figure 3A). AIstain is employed as a virtual channel in the Olympus ScanR software. After acquisition, there is no need of complex gating. However, there is the possibility to gate for subsets of cells based on special morphological features or size and complexity of AIstain-based cell segmentation to achieve a deeper readout of the assay if necessary.

Running the live cell HCA of phagocytosis in primary microglia using AIstain shows a time-dependent uptake of pHrodo phagocytosis beads (Figure 3B). Furthermore, the treated microglia show reduced phagocytic activity with a reduced maximum uptake compared to the control (Figures 3B and 3C). Each data point shows the mean of approximately 5,000 single cells per single replicate. To conclude, AIstain allowed us to reduce the time of sample preparation, concomitant with gaining a higher level of accuracy in the experiment. Furthermore, the phagocytic activity was not influenced by additional substances or dyes other than our treatment, leading to a better translatability of the results.

### Other cell types can also be reliably detected by AIstain

**Figure 4:**
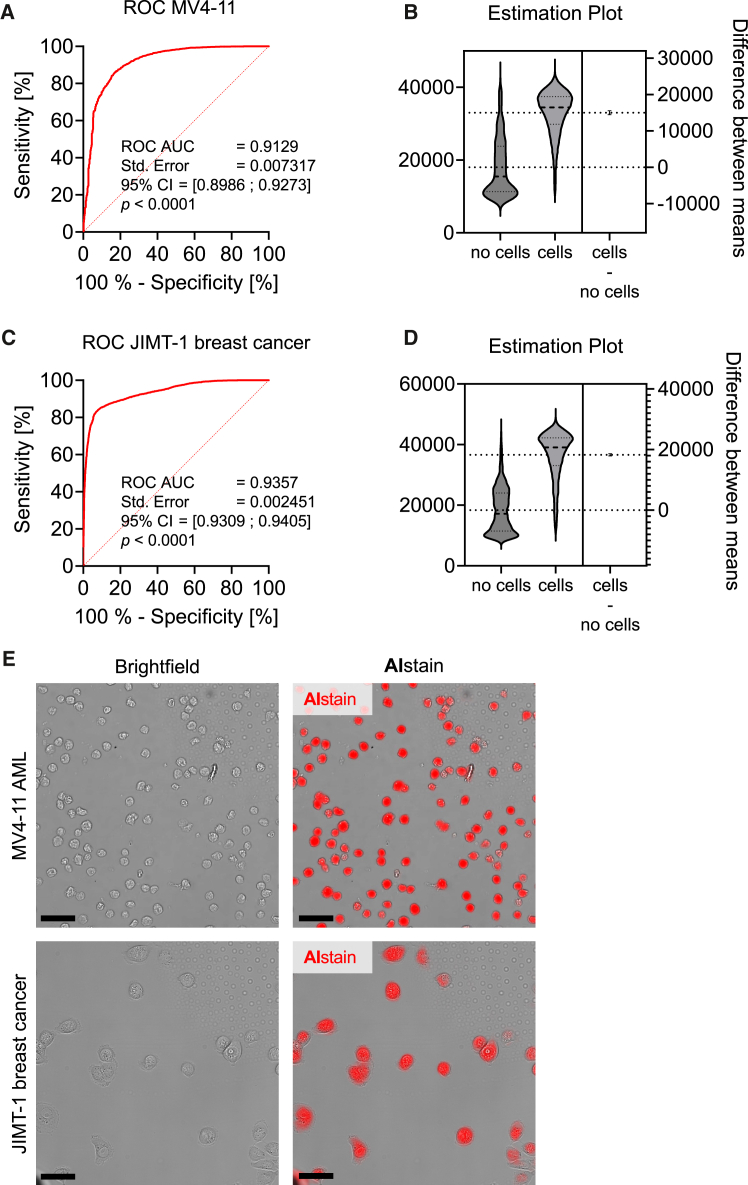
The neural network can also be applied on other cell types (A) Receiver operator curve showing neural network performance on the MV4-11 AML cell line. Dataset contains 8.693 entries; 95% confidence interval was chosen with Wilson/Brown method. (B) Estimation plot showing the distribution of the neural network prediction value (16-bit scale, the values 0, 32,767, and 65,535 correspond to classification probabilities of 0%, 50%, and 100%) for the classes no cells and cells in MV4-11 AML cells. The neural network prediction value resembles the probability of the recognized object being a cell. The difference between means shows the difference of the mean prediction value in the class no cells compared to difference of the mean prediction value in the class cells. The higher this value is, the better the segregation of the two classes. (C) Receiver operator curve showing neural network performance on the JIMT-1 breast cancer cell line. Dataset contains 9.796 entries; 95% confidence interval was chosen with the Wilson/Brown method. (D) Estimation plot showing the distribution of the neural network prediction value (16-bit scale, the values 0, 32,767, and 65,535 correspond to classification probabilities of 0%, 50%, and 100%) for the classes no cells and cells in JIMT-1 breast cancer cells. The neural network prediction value resembles the probability of the recognized object being a cell. The difference between means shows the difference of the mean prediction value in the class no cells compared to difference of the mean prediction value in the class cells. The higher this value is, the better the segregation of the two classes. (E) Representative images showing deep learning artificial *in silico* staining (red) of the MV4-11 AML cell line (upper panel) and the JIMT-1 breast cancer cell line (lower panel). Scale bars: 50 μm.

We were further interested in whether AIstain detects and *in silico* stains other cell types as well. For this purpose, we imaged unstained MV4-11 acute myeloid leukemia (AML) cells. The performance of AIstain on AML cells was not as good as on microglia, but we were still able to achieve a good AUC ROC of 0.9129 (Figure 4A) with a good probability of segregation of cells vs. no cells (Figure 4B), indicating a reliable trade-off between true-positive and false-positive detections.

Since both, microglia and MV4-11 AML cells, are of myeloid origin, we aimed to evaluate our deep learning model on cell types of other origins as well. To do so, we used epithelial JIMT-1 breast cancer cells exhibiting a clearly different morphology than microglia or AML cells. However, despite the different origin of the cells, ROC analysis revealed a high performance of AIstain with an AUC ROC of 0.9357 (Figure 4C), with an even better detection rate than on AML cells, indicating a broad applicability of AIstain beyond microglia. This is further supported by a clear segregation of the probabilities (Figure 4D). To sum up, AIstain can be transferred to other cell types as well without a relevant loss of accuracy of artificial *in silico* staining (Figure 4E).

### The U-Net-based approach AIstain can be translated to other research questions

**Figure 5:**
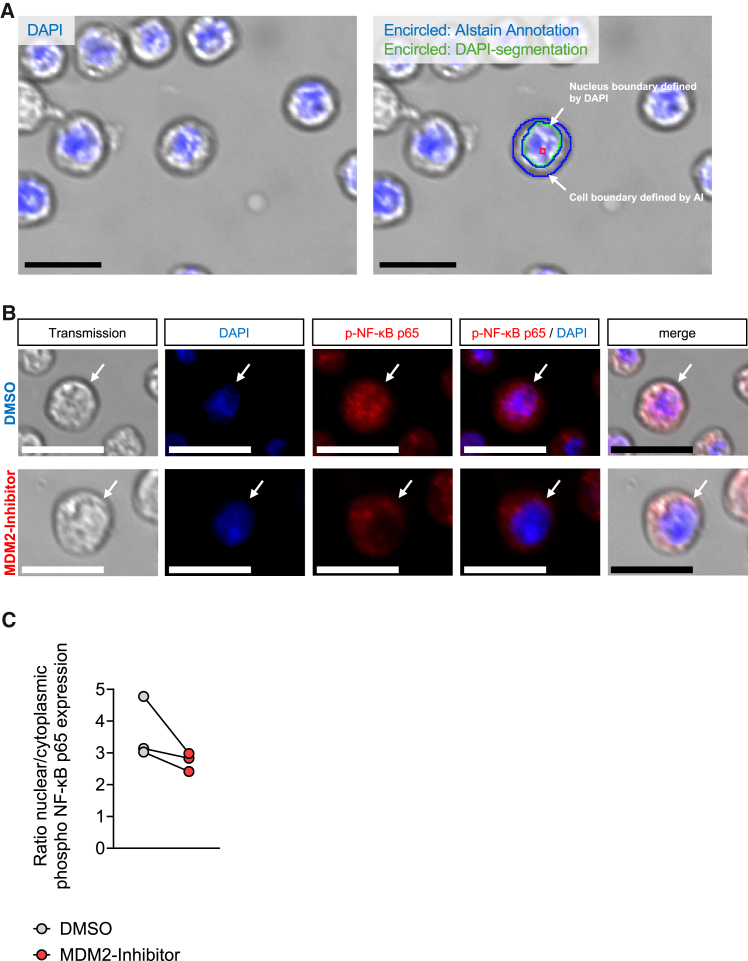
The neural network can be translated to other research questions (A) Representative images showing deep learning cell detection of the E2A-PBX B-ALL cell line. (Left) Bright-field image merged with DAPI for nuclear staining. (Right) Overlaid cell detection with nucleus encircled in green and cytoplasm encircled in blue. Scale bars: 20 μm. (B) Representative images showing immunofluorescence staining for phospho NF-κB p65 (red) and DAPI (blue) of E2A-PBX B-ALL cells treated with either DMSO (upper panel) or kinase inhibitor (lower panel). Scale bars: 20 μm. (C) Scatter dot plot showing ratio of nuclear vs. cytoplasmic phospho NF-κB p65 expression in E2A-PBX B-ALL cells treated with either DMSO or MDM2 inhibitor.

We were further interested to investigate whether our U-Net based approach could also be used for other applications than phagocytosis of microglia, since we showed a reliable detection of other cell types by our model. To do so, we addressed the important question in biological research and neuroinflammation: whether the intracellular localization of proteins or transcription factors such as nuclear factor kappa-light-chain-enhancer of activated B cells (NF-κB) changes in disease or upon treatment. NF-κB is a transcription factor that regulates genes involved in inflammation, immune response, cell survival, and apoptosis. NF-κB resides in the cytoplasm in steady state, but upon inflammatory signaling, NF-κB gets activated by phosphorylation.^32^ The p65 (RelA) subunit is a key component of the NF-κB complex.^32^ The phosphorylation of p65 (at specific sites like Ser536 or Ser276) is a critical modification that enhances its activity. This modification guides NF-κB to translocate to the nucleus, bind genomic regions in the binding site, and activate transcription of target genes.^33^^,^^34^ Thus, the nuclear translocation of NF-κB serves as a surrogate marker for NF-κB pathway activity and inflammatory signaling. Vice versa, reduced nuclear localization indicates reduced inflammatory signaling.^35^^,^^36^ Phospho NF-κB p65 expression refers to the amount of phosphorylated p65 subunit present in the cell, typically assessed through western blotting, immunofluorescence, or ELISA. Its measurement helps researchers to study NF-κB activation and its role in disease progression or treatment responses. We used the E2A-PBX B-cell acute lymphoblastic leukemia cell line as an easily accessible B cell model and treated them with a MDM2 inhibitor, fixed them, and stained them with DAPI and anti-phospho NF-κB antibody. Common assays would also require a membrane staining to distinguish nuclear and cytoplasmic signal in fluorescence microscopy; however, we used our U-Net-based approach AIstain to define the cytoplasm of the cells thereby avoiding the need for further staining (Figure 5A). This opens one more fluorescence channel for other stainings in the same cells, for example, to investigate protein-protein interactions. In our model, we quantified cytoplasmic vs. nuclear localization of NF-κB and could show that MDM2 inhibition reduces the nuclear translocation of NF-κB, indicating reduced NF-κB activity and inflammatory signaling (Figures 5B and 5C). These results further support that AIstain demonstrates a broad applicability beyond detecting microglia in phagocytosis assays.

### Conclusions

Studying microglial phagocytosis is essential to fully understand neuroinflammatory diseases^4^ and to develop new therapeutic strategies.^5^ Phagocytosis assays often employ flow cytometry, live cell imaging, or HCA,^6^^,^^7^ requiring cell labeling^8^ to allow cell detection by subsequent image analysis. However, cell labeling is known to affect the phagocytic behavior of macrophages,^9^ pointing out the need for novel methods without cell labeling.

Our study demonstrates the efficacy of using the deep learning-based approach AIstain to investigate microglial phagocytosis in live cell image cytometry or HCA. By leveraging the capabilities of CNNs, particularly the U-Net architecture, we were able to significantly improve the accuracy and efficiency of microglial detection and analysis compared to fluorescence cell staining as well as common segmentation tools such as Cellpose 3 and SAM2. Cellpose 3 and SAM2 are well-established tools for image segmentation and have demonstrated their advancements before.^29^^,^^30^^,^^31^ However, both tools are not yet supported in the Olympus ScanR software making their applicability to high-throughput imaging using the Olympus ScanR environment uncomfortable as every single image has to be analyzed in a stand-alone image analysis software such as QuPath or via python and R software solutions, which required programming skills or access to a bioinformatics unit.

The decision to employ a U-net architecture, as implemented in AIstain, was driven by the objective of providing a solution that is both readily applicable and yet potent in its capacity to utilize contemporary deep-learning architectures for the study of phagocytosis in imaging-based assays. This was facilitated by Olympus ScanR analysis software, which supports the U-Net model. Other models are currently not supported by the software. By using the U-Net architecture, we ensured the broad applicability on existing Olympus ScanR systems and the easy use by scientists without prior expertise in bioinformatics. Furthermore, U-Net excels at segmenting complex biological structures, even in challenging conditions like varying cell sizes,^37^^,^^38^ making it especially reliable for detecting microglia as they exhibit dynamic morphological changes.^4^

The implementation of AIstain in the Olympus ScanR system showed substantial improvements in detection performance. Using 5-fold cross-validation, we demonstrate the robustness of our model to generalize the entire dataset. The U-Net-based segmentation achieved high AUC ROC values, highlighting its strong capability in labeling microglia. Importantly, AIstain maintained high accuracy even in the presence of increased background with reduced signal-to-noise ratio due to phagocytosis beads and cell debris, which typically challenges cell segmentation^39^^,^^40^ and fluorescence staining methods, even in flow cytometry.^41^ Terho et al.^42^ proposed an additional fluorescence staining in flow cytometry to exclude cell debris,^42^ underscoring the problems in cell staining.

When comparing our described approach to the live cell staining (CellTracker), it not only increased the true-positive but also reduced the false-positive rate at both initial and later time points, indicating that the approach provides more reliable and consistent cell detection over extended periods of imaging, which is crucial for dynamic studies^43^^,^^44^ such as time-lapse phagocytosis assays. Moreover, we could show an enhanced performance of AIstain in microglia detection compared to the widely used U-Net-based segmentation tool Cellpose 3 and the latest segmentation tool SAM2, which is not U-Net based, but built up on a Hiera architecture.^30^^,^^31^ These results underline the advancement of AIstain for microglia cell detection on the Olympus ScanR system. Although the required time of analysis is higher, the trade-off is justified by the substantial gains in accuracy and reliability. It should also be emphasized that the analysis runtime is highly dependent on the computer hardware and network environment.

A significant advantage of using the AIstain-based approach is its ability to improve the workflow for live cell image cytometry. Traditional methods requiring live cell dyes, cell fixation,^45^^,^^46^ or at least a nuclear staining^47^ are often associated with cytotoxicity^25^ and phototoxicity,^48^ which can affect not only cell viability but also cell motility and experimental outcomes.^24^ Our label-free U-Net-based approach mitigates these issues, allowing for more accurate and physiological analyses. The reduction of sample preparation time by avoiding staining procedures further enhances the practicality and accessibility of this technique for high-throughput studies. The ability to monitor phagocytic activity in real time without the need for toxic dyes allows for more accurate assessment of cellular functions and interactions.

AIstain also demonstrated versatility in detecting other cell types beyond microglia. The model performed well on AML cell line and JIMT-1 breast cancer cell line, which enables researchers to implement the provided neural network in other applications besides phagocytosis assays as we demonstrated for NF-κB signaling studies as well. This suggests that the AIstain-based approach can be generalized to a wide range of cell types and research questions, underscoring its potential for broad applicability in cell biology research.

Despite the promising results, further optimization of the neural network design is necessary to address challenges associated with different cell morphologies and experimental conditions. Future studies should focus on refining the model to enhance its accuracy across diverse cell types and improving the speed of data analysis to make the approach even more efficient. Moreover, integrating our model with other advanced imaging techniques like high-resolution imaging and expanding its capabilities to analyze additional cell behaviors, states, and interactions will provide deeper insights into cellular processes and disease mechanisms.

In conclusion, our study introduces the robust U-Net AIstain for the accurate and efficient analysis of microglial phagocytosis in live cell image cytometry. By eliminating the need for potentially toxic staining procedures and providing high-throughput, single-cell resolution analysis, our study presents an easy-to-use approach, leading to a significant advancement in the field of neurobiological research. The broad applicability of AIstain to other cell types further enhances its potential as a versatile tool for cell biology studies, paving the way for more detailed and accurate studies on cellular functions and disease pathogenesis.

### Limitations of the study

The described method was originally developed for the analysis of microglia as adherent cells; however, the neural network showed good performance on suspension cells like the MV4-11 AML cell line as well, but further validation is needed for other experimental setups using non-adherent cells. Cell death within the sample can further significantly compromise data quality, as dead cells may be detected and analyzed by the neural network, leading to inaccurate results. Therefore, maintaining cell viability during experiments is crucial. The major limitation of our provided method is the dependency on the Olympus ScanR system. This reliance on specific hardware and software limits the generalizability of the method, making it challenging to adapt it to other imaging systems without significant modifications. Furthermore, processing power can significantly impact the time required to complete the analysis. The deep learning model employed in this method is computationally intensive, requiring substantial resources that may not be readily available in all research settings.

## Resource availability

### Lead contact

Further information and requests for resources and reagents should be directed to and will be fulfilled by the lead contact, Robert Zeiser (robert.zeiser@uniklinik-freiburg.de).

### Materials availability

This study did not generate new unique reagents.

### Data and code availability

• The training and validation datasets supporting the conclusions of this article are available on Zenodo: Zähringer, Alexander (2025), AIstain: Enhancing microglial phagocytosis analysis through deep learning – Validation Dataset,” Zenodo, https://doi.org/10.5281/zenodo.15689727, and Zähringer, Alexander (2025), AIstain: Enhancing microglial phagocytosis analysis through deep learning – Training Dataset,” Zenodo, https://doi.org/10.5281/zenodo.15532325.
• The neural network supporting the conclusions of this article and for free usage are available on Mendeley Data^49^ Zähringer, Alexander (2024), “Enhancing microglial phagocytosis analysis through deep learning: A U-Net based approach,” Mendeley Data, V1, https://doi.org/10.17632/czh96my8cf.1.
• Any additional information required to reanalyze the data reported in this paper is available from the lead contact upon request.

## Acknowledgments

We thank the Lighthouse Core Facility for their support with imaging. Lighthouse Core Facility is funded in part by the 10.13039/501100021729Medical Faculty, University of Freiburg (project numbers 2023/A2-Fol and 2021/B3-Fol) and the 10.13039/501100001659DFG (project number 450392965). This study was supported by the 10.13039/501100001659Deutsche Forschungsgemeinschaft ([DFG] 10.13039/501100001659German Research Foundation) – SFB-1479 – Project ID: 441891347 (P01 and S02 to R.Z.), Project-ID 259373024 – TRR 167 (to R.Z.), and 10.13039/501100001659DFG: RU5659 TARGET-MPN: ZE 872/6-1 (TP 7); the European Union: EU Proposal n°ERC-2022-ADG Project: 101094168 – AlloCure (ERC Advanced Grant to R.Z.); ERA-NET Transcan – PIXEL (to R.Z.); ERA-NET Transcan – SmartCART (to R.Z.); the Germany's Excellence Strategy (CIBSS – EXC-2189 – Project ID 390939984 to R.Z.); the MOTI-VATE program of the Medical Faculty, Albert-Ludwigs-University of Freiburg (A.Z.); the 10.13039/501100005972Deutsche Krebshilfe (grant number 70114655); and the Jose-Carreras Leukemia foundation grant number DJCLS 09R/2022 (R.Z.), EU project: Project 101119855 – exTra, 10.13039/100005189Leukemia and Lymphoma Society (LLS Grant ID: 7030-23 to R.Z.). This project was also supported by EU project: Project 101119855 – exTra. T.W. was supported by the Berta-Ottenstein clinician scientist fellowship of the Medical Faculty, Albert-Ludwigs-University of Freiburg.

## Author contributions

A.Z. performed most of the experiments, helped to develop the overall concept, analyzed data, and wrote the manuscript. J.M.V., T.W., P.S., and M.F. helped with the experiments, contributed to critical analysis of the data, and helped improve the manuscript. F.I. helped with bioinformatic evaluation of the U-Net and analysis of the data. R.Z. supervised the experiments, analyzed data, and helped write the manuscript.

## Declaration of interests

R.Z. has received honoraria from Novartis, Incyte, Sanofi, Medac, Neovii, and Mallinckrodt.

## Declaration of generative AI and AI-assisted technologies in the writing process

During the preparation of this work, the authors used chatgpt.com (openAI) in order to re-phrase paragraphs and check for grammar. After using this tool/service, the authors reviewed and edited the content as needed and take full responsibility for the content of the publication.

## STAR★Methods

### Key resources table

**Table:** 

REAGENT or RESOURCE	SOURCE	IDENTIFIER
**Chemicals, peptides, and recombinant proteins**		

FluoroBrite™ DMEM	Gibco™ Thermo Fisher	Cat#A1896701
DMEM/F-12 (1:1) + GlutaMAX Supplement	Gibco™ Thermo Fisher	Cat#31331093
RPMI 1640 medium	Gibco™ Thermo Fisher	Cat#11875093
Penicillin/streptomycin	Gibco™ Thermo Fisher	Cat#15140-122
murine GM-CSF	Homemade	N/A
Dulbecco’s Phosphate Buffered Saline (PBS)	Sigma-Aldrich	Cat#D8537-500ML
Fetal Bovine Serum South America (FCS)	anprotec	Cat#AC-SM-0027
Dimethyl sulfoxide (DMSO)	Carl Roth GmbH + Co. KG	Cat#A994.2
FICZ	MedChemExpress	Cat#HY-12451
4% Formaldehyde	Liquid Production GmbH	Cat#FN-5000-4-1
CellTracker™	Invitrogen™ Thermo Fisher	Cat#C7025
Hoechst 33342	Sigma-Aldrich	Cat#B2261-25MG
HDM201	MedChemExpress	Cat#HY-18658
Albumin Fraktion V, US-Origin ≥96%, für die Biochemie und Molekularbiologie (BSA)	Carl Roth GmbH + Co. KG	Cat#3854.3
HISTOPRIME Normal Goat Serum, w/Preservative (0.08% NaN3), Non Sterile	Biozol/Linaris	Cat# LIN-ENG9010-10
Triton™ X-100	Sigma-Aldrich	Cat# X100-5ML
Rabbit anti-phospho NF-κB Ser536	Invitrogen™ Thermo Fisher	Cat#MA5-15160; RRID: AB_10983078
Goat-*anti*-rabbit IgG (H + L) AlexaFluorPlus647 secondary antibody	Invitrogen™ Thermo Fisher	Cat#A-32733; RRID: AB_2633282
4′,6-Diamidin-2-phenylindol-dihydrochlorid (1mg/ml) (DAPI)	Sigma-Aldrich	Cat# D8417

**Critical commercial assays**		

pHrodo™ BioParticles™ Conjugates for Phagocytosis and Phagocytosis Kit, for Flow Cytometry – Deep Red	Invitrogen™ Thermo Fisher	Cat#P35360

**Deposited data**		

AIstain training and validation dataset	This paper	ZENODO https://doi.org/10.5281/zenodo.15689727 and https://doi.org/10.5281/zenodo.15532325

**Experimental models: Cell lines**		

Primary murine microglia obtained from C57BL/6 mice, female, 6–10 weeks old	Generated by the authors of this paper	N/A
JIMT-1 breast cancer cell line, human, female	Tanner et al.^50^	JIMT-1; RRID: CVCL_2077
MV4-11 AML cell line, human, male	Beverly et al.^51^	MV4-11; RRID: CVCL_0064
E2A-PBX B-ALL cell line, murine	Dr. Macell, Standford University, USA	N/A

**Experimental models: Organisms/strains**		

C57BL/6 mice female, wildtype, 6–10 weeks old	CEMT University Medical Center Freiburg	N/A

**Software and algorithms**		

Olympus ScanR Acquisition 3.4.1 or 3.5 (or higher)	Olympus Life Science Solutions	https://www.olympus-lifescience.com/de/support/downloads/%20/
Olympus ScanR Analysis 3.4.1 or 3.5 (or higher)	Olympus Life Science Solutions	https://www.olympus-lifescience.com/de/support/downloads/%20/
Trained Neural Network AIstain	This paper	Zähringer^49^
AIstain Sample Assay	This paper	Zähringer^49^
Cellpose 3	GitHub	Cellpose main repository
SAM2	GitHub/Meta	QuPath extension SAM
QuPath v0.5.1	GitHub	QuPath installer
GraphPad Prism	GraphPad Software, San Diego, CA	GraphPad Prism

**Other**		

Olympus ScanR IX-83 high-throughput microscope	Olympus Life Science Solutions	Olympus-lifescience ScanR
Bath sonicator	N/A	N/A
CellVis 96 Well glass bottom plate with high performance #1.5 cover glass	Cellvis	Cat# P96-1.5H-N

### Experimental model and study participant details

#### Primary microglia

Animal protocols (Protocol numbers: X-20/07A, X-20/06K, X-15/10A, G-23/075, G-17/) were approved by the Regierungspräsidium Freiburg, (regional council), Germany (Federal Ministry for Nature, Environment and Consumers Protection).

Primary microglia were derived from female C57BL/6 mice (purchased either from Janvier Labs (France) or from the local stock of the animal facility at the University of Freiburg. Mice were maintained at the animal facility at the University of Freiburg. Mice were used between 6 and 14 weeks of age. After perfusion and removal of the brain, mixed glial culture was set up by enzymatic digestion and gradient centrifugation using PERCOLL. Cells were cultured in DMEM/F-12 (1:1) + GlutaMAX Supplement (Gibco #31331093) with 10% FCS (anprotec #AC-SM-0027), 100μg/ml penicillin/streptomycin (Gibco #15140-122) and 5ng/ml murine GM-CSF at 37°C and 5% CO_2_ fully humified atmosphere. Mature primary microglia lifted off the plate and were collected for further experiments. Medium was changed twice a week.

#### JIMT-1 breast cancer cell line

The JIMT-1 breast cancer cell line is a well-characterized human breast cancer model, particularly noted for its resistance to trastuzumab (Herceptin). The cell line was established from a 62-year-old female patient with HER2-positive breast cancer who had received trastuzumab-based therapy as described earlier by Tanner et al.^50^

The cells were cultured in RPMI-1640 with 10% FCS (anprotec #AC-SM-0027) and 100μg/ml penicillin/streptomycin (Gibco #15140-122) at 37°C and 5% CO_2_ fully humified atmosphere.

#### MV4-11 AML cell line

The MV4-11 cell line is a widely used human AML cell line derived from the peripheral blood of a 10-year-old male patient with biphenotypic B-myelomonocytic leukemia as described earlier.^51^

The cells were cultured in RPMI-1640 (Gibco #11875093) with 10% FCS (anprotec #AC-SM-0027) and 100μg/ml penicillin/streptomycin (Gibco #15140-122) at 37°C and 5% CO_2_ fully humified atmosphere.

#### E2A-PBX B-ALL cell line

The E2A-PBX B-cell acute lymphatic leukemia murine cell line was kindly provided by Dr. Mackall, Standford University, USA and derived from E2A-PBX1 transgenic mice crossed with mice developing spontaneous pre-B-ALL.

The cells were cultured in RPMI-1640 (Gibco #11875093) with 10% FCS (anprotec #AC-SM-0027) and 100μg/ml penicillin/streptomycin (Gibco #15140-122) at 37°C and 5% CO_2_ fully humified atmosphere.

All cell lines were continuously tested for mycoplasma contamination.

### Method details

#### Phagocytosis assay

A total of 10,000 primary microglia per well were plated in 96-well glass bottom plate and treated for 48h with 500nM FICZ or DMSO. Medium was removed and FluoroBrite medium (Gibco #A18967-01) was added. Cells were incubated in 100μL FluoroBrite (Gibco #A18967-01) medium, supplemented with 10% FCS and 500nM FICZ or DMSO on an Olympus ScanR IX-83 high-throughput microscope using an UPLSAPO 20x/0.75 objective in 37°C 5% CO_2_ atmosphere for live cell imaging. pHrodo deep red bioparticles (Invitrogen #P35360) were added according to the manufacturer’s instructions and cells were imaged for 3.5 h every 15 min. The trained neural network was used later as a virtual channel to define the cytoplasm by threshold-based segmentation of the probability map as shown in Figure S3A. Total intensity of pHrodo was used as the readout. A detailed step-by-step protocol is provided in Zähringer et al.^52^ An application of the phagocytosis assay was previously described in Zähringer et al.^4^

#### Neural network training

The neural network was trained from scratch without prior pre-training on generic image datasets. For neural network training, 10,000 primary microglia per well were stained with 10 μM CellTracker (Invitrogen #C7025) and 1 μg/mL Hoechst 33342 (Sigma-Aldrich #B2261-25MG) in FluoroBrite medium (Gibco #A18967-01) for 30 min prior to imaging. Cells were washed three times with FluoroBrite medium (Gibco #A18967-01). Cells were incubated in 100 μL FluoroBrite medium, supplemented with 10% FCS on an Olympus ScanR high-throughput microscope in 37°C 5% CO_2_ atmosphere for live cell imaging. A total of 8 wells were acquired for 3.5 h every 15 min. Using Olympus ScanR Analysis 3.4.1, cells were gated by Hoechst expression and CellTracker-positivity (Figures S1A and S1B). Gated cells were used as the input for the brightfield channel for the deep learning. The brightfield images were paired with the fluorescence images. The alignment was ensured by the Olympus ScanR system as both channels were acquired together and resemble different masks of one image. The fluorescence masks was used to outline the cells by gating in the Olympus ScanR software as described below. We used a typical training-validation split of 80/20%. Thereby we ensured, that the U-Net has not seen the images used for validation before. The corresponding datasets can be downloaded on Mendeley Data/Zenodo. U-net was selected as network architecture with balanced data sampler, normalization by local contrast and semantic object handling. Semantic object handling describes that the U-Net classifies each pixel as belonging to a specific class (e.g., “cell” or “background”). However, it does not inherently distinguish between different individual cells, meaning that overlapping or clustered cells may be merged into a single segmented region. This is not a problem for further analyses as the Olympus ScanR software segments the output probability map and annotates each single object with its own object ID (visualized by different colors) as shown in Figure S3A, allowing the extraction of single cells even though the U-Net used semantic object handling. In addition, 90-degree rotations and mirroring were used for dynamic geometric augmentation without image processing augmentation. The standard augmentations 90-degree rotations and mirroring are chosen because they do not introduce artifacts (e.g., through interpolation) in most contrast methods. Loss has been taken into account by Softmax cross entropy (standard loss as published before by Ronneberger et al.^25^). AdamW optimizer was used. 25,000 iterations were done. Learning rate was set to 10^−4^, Batch size was set to 2. No dropout or weight decay was used, but online validation using 10% of the training dataset was carried out by the Olympus ScanR software (Jaccard index) as described earlier.^53^^,^^54^ Together with generating checkpoints during the training, this allowed us to avoid overfitting (early stopping of the training). Training performance was monitored using the pixel-wise Jaccard index. The neural network was trained in the environment of the Olympus ScanR Analysis 3.4.1 software. The trained neural network is available via Mendeley Data (Zähringer, Alexander (2024), “Enhancing microglial phagocytosis analysis through deep learning: A U-Net based approach”, Mendeley Data, V1, https://doi.org/10.17632/czh96my8cf.1). The training and validation datasets supporting the conclusions of this article are available on Zenodo (Zähringer, Alexander (2025), AIstain: Enhancing microglial phagocytosis analysis through deep learning – Validation Dataset”, Zenodo, https://doi.org/10.5281/zenodo.15689727 and Zähringer, Alexander (2025), AIstain: Enhancing microglial phagocytosis analysis through deep learning – Training Dataset”, Zenodo, https://doi.org/10.5281/zenodo.15532325). The U-Net model be easily imported in the Olympus ScanR Analysis software as described in Zähringer et al.^52^

We evaluated sensitivity and specificity by manual annotation. We annotated cells in the brightfield images (ground truth) and overlaid the AIstain or CellTracker stain and calculated the percentage of detected cells’ coincidence.

5-fold cross-validation was carried out to demonstrate generalizability and robustness of the U-Net to unseen data. Pixel-wise Jaccard Index was used to monitor training dynamics. The training dataset was divided in 5-folds and 5 single U-Nets were trained on 5 different training datasets consisting of 4 of the 5-folds. The fold which was not part of the training dataset was used for validation. The performance metrics are shown in Figures S2A and S2B.

The following hardware was used for training and validation of AIstain.

- Processor: Intel Xeon W-2255 CPU 3.70 GHz.
- RAM: 64 GB.
- System: 64 Bit.
- Graphic card: NVIDIA Quadro RTX 4000.
- Dataset was saved on institutional network server.

Systems with less computing power should be avoided as downstream analyses of the phagocytosis assay besides the U-Net are computationally intensive.

#### U-net architecture

(1) Encoder Path (Downsampling):
  • The encoder consists of 5 downsampling stages.
  • Each stage includes:
    ○ Two convolutional layers per stage.
    ○ A pooling operation after every stage.
  • The number of feature maps increases progressively.
  • Filter sizes: Convolutional layers have 3 × 3 filters.
  • Strides: Strides are 1 for convolutional layers and 2 for pooling layers.
(2) Decoder Path (Upsampling):
  • The decoder mirrors the encoder with 5 upsampling stages.
  • Each upsampling stage consists of:
    ○ A transposed convolution (upsampling layer).
    ○ Two convolutional layers after upsampling.
    ○ Skip connections from corresponding encoder layers (concatenation).
  • Filter sizes: 3 × 3 for convolutional layers.
  • Strides: Stride 2 for transposed convolutions (upsampling), stride 1 for convolutional layers.
(3) Bottleneck (Bridge):
  • This layer connects the encoder and decoder.
  • Includes two convolutional layers before transitioning to the upsampling path

We used ReLU due to its robustness to introduce non-linearity within each of the layers and its previous application as activation for a wide range of deep-learning applications. We did not compare ReLU to other activation functions.

The implementation of skip connections involved the use of four such connections, with slicing and concatenating techniques employed to facilitate this. The “Slice” operation extracts relevant portions of the encoder’s feature maps, and the “Concat” operation merges them with the decoder’s feature maps. This enables the decoder to utilize both high-level, abstract features (from deeper layers) and low-level, detailed features (from earlier layers), thereby enhancing segmentation performance.

The configuration of the output layer is as follows: The Softmax function is employed for the purpose of pixel-wise classification.

The total number of parameters in the model is 7,237,380.

The code of the U-Net is available on Mendeley Data and can be visualized using by e.g., Netron (https://netron.app/).

#### Gating strategy

The scan was analyzed using Olympus ScanR analysis 3.4.1 software. A new assay was set up with nuclei-segmentation as main object. The nucleus was segmented based on the intensity of Hoechst. Watershed was enabled (minimum object size 100 pixels, maximum object size 1,000,000 pixels) and threshold for Hoechst intensity was set to 6,147. Subobjects were defined for cytoplasm using an individual segmentation-threshold for CellTracker staining (threshold = 7,485). The cytoplasm was defined on a main object mask which means that the cytoplasm is only defined in a region where a nucleus was segmented in the prior step which minimizes artifacts. The following parameters were defined for all objects: Area, Perimeter, Circularity, total intensity of Hoechst/CellTracker, X and Y position and well. Each single detection will generate one dot in a dot plot which resembles biaxial dot plots in flow cytometry.

Nuclei were gated as shown in Figure S1A. The nuclei populations (R01) were determined based on area (X axis) and circularity (Y axis) of the main object (nucleus, Hoechst signal). These cells inside R01 were further gated (R02), based on their CellTracker segmentation (thresholding as described above). Cells were gated as shown in Figure S1A. The population (R02) was determined based on area (X axis) and circularity (Y axis) of the subobject (cytoplasm). The combination of Hoechst signal and CellTracker signal for this gate allowed us to remove cell debris or dying cells.

R02 was used as the input gate for the U-Net training. The gates were counterchecked by visualization of the included cells (displaying the “Gallery” of the gates as shown in Figure S1B). This was also used to fine-adjust the gating. The cells shown in the “Gallery” are randomly selected by the Olympus ScanR software and are representative of the population inside the gate.

For the cell gating of the training dataset, overlapping cells were divided by water-shedding as described above. However, cell overlapping was rare, since only plated 10,000 cells were plated per well, which corresponds to the setup of the phagocytosis-assay experiment that to allows monitoring of cell migration and formation of cell protrusions. Poorly segmented cells will likely have non-physiological cell parameters regarding Area and Circularity, so that these cells appear as cell debris and were excluded by the gating described above prior to training.

All cells plated in the well that exhibited physiological cell parameters (Area, Circularity, Hoechst signal and CellTracker signal) were included in the training/cell population. This ensures that the gated cells are representative of the population. The gated cells correspond to the cell population. Microglia cells themselves are heterogeneous since they change morphology continuously which allowed a training the U-Net on different cell shapes at the same time. This is underlined by the gate R02 in Figure S1A which shows a heterogeneous cell population along “Area”. This effect was further increased by the time laps experiment setup.

#### Definition of ground truth

The ground truth was determined by manual annotation. Additionally, we checked for false positive classification by re-gating for internal control to increase accuracy, as follows:

All AIstain-detections were gated for Area and Circularity of the cell body. We gated on all the detections which exhibit parameters that are typical for cells. This allows us to detect cell debris which is classified as cells since it shows unphysiological parameters (Area, Circularity etc.). All detections outside this gate were likely false positive classifications and were counterchecked by visualization and inspection. The gating is shown in Figure S5A. The cross-hair (red) far outside the gate (yellow) points to a false positive classification. The correlating object in the image is shown in Figure S5B. The green line marks the corresponding AIstain detection, which clearly demonstrates that the indicated object is not a cell. This shows that the parameters Area and Circularity are good in the discrimination between cells and debris. Furthermore, we checked for false negative classification by re-gating. Nuclei were segmented by thresholding and gated as described above and shown in Figure S5C. For each segmented nucleus, we checked for the overlaying classification of the AIstain (Max. intensity cytoplasm probability). False negative classification would result in a segmented nucleus without an AIstain “cell-detection” as shown in Figures S5C and S5D. False negative classifications were counterchecked manually as well.

To fine-adjust the gates, we visualized the gallery of the gate to ensure a high accuracy as shown in Figure S1B.

#### Cellpose 3

Cellpose 3 was used in QuPath v0.5.1. Cellpose 3 was installed according to the instructions provided in the Cellpose main repository on GitHub. The script used to segment the cells in the brightfield images is shown in Methods S1. Performance was evaluated by manual annotation and counting.

#### SAM2

SAM2 was installed as a QuPath plugin in QuPath v0.5.1 as described before.^55^ We followed the detailed instructions provided on GitHub QuPath extension SAM.

The images were imported into QuPath and each single images was analyzed by running the SAM2 plugin with the settings shown in Methods S2:

Performance was evaluated by manual annotation and counting.

#### Image cytometry of living cells

10,000 cells per well were incubated in 100 μL FluoroBrite medium, supplemented with 10% FCS on Olympus ScanR high-throughput microscope (UPLSAPO 20x/0.75 objective) in 37°C 5% CO_2_ atmosphere for live cell imaging. The trained neural network was used as a virtual channel to define the cytoplasm. To analyze the performance metrics of the neural network, cells were manually annotated. Manual annotations and artificial staining by the neural network were either counted or a ROC curve was plotted.

#### Image cytometry of E2a-PBX B-ALL cells

100,000 cells per well were plated and treated with 0.02 μM HDM201. Afterward, cells were fixed using 4% formaldehyde for 5 min, permeabilized and blocked using 5% BSA +5% Goat serum +0.1% Triton X-100 in PBS for 10 min. Cells were stained for 1 h with the primary antibody (Rabbit anti-phospho NF-κB Ser536 #MA5-15160, Invitrogen ThermoFisher) and for 1 h with Goat-*anti*-rabbit IgG (H + L) AlexaFluorPlus647 secondary antibody (#A-32733, Invitrogen ThermoFisher). DAPI was added for 5 min at 1:10,000. Cells were washed three times for 5 min in PBS and then imaged on the Olympus ScanR high-throughput microscope (UPLSAPO 20x/0.75 objective). The trained neural network was used as a virtual channel to define the cytoplasm.

### Quantification and statistical analysis

#### Statistics and reproducibility

The experiments were performed in a non-blinded fashion. Data was tested for normality using the Kolmogorov-Smirnov test. For normally distributed data, an unpaired 2 tailed Student’s t test was applied for 2 groups. If the data did not meet the criteria of normality, the two-tailed Mann-Whitney U test was applied. If more than 2 groups were analyzed, the Kruskal-Wallis-Test was used in case of nonparametric distribution. One-way ANOVA was performed in the case of normally distributed data.

Statistical analysis was done using GraphPad Prism (GraphPad Software; San Diego, CA). Data are presented as mean and s.e.m. (error bars). *p*-value <0.05 was reported as statistically significant. The data collection was not randomized. Data collection and analysis were not performed blind to the conditions of the experiments.

## Supplemental information

Document S1. Figures S1–S5 and Methods S1 and S2

Document S2. Article plus supplemental information
