# Supplementary material for: AIstain: Enhancing microglial phagocytosis analysis through deep learning
Source: Cell Rep Methods. 2025 Oct 17;5(11):101207. doi: 10.1016/j.crmeth.2025.101207 (PMC12664894; doi:10.1016/j.crmeth.2025.101207)
Supplement: Document S1. Figures S1–S5 and Methods S1 and S2 [file mmc1.pdf]

Cell Reports Methods, Volume *5*

Supplemental information

AIstain: Enhancing microglial phagocytosis analysis through deep learning

Alexander Zähringer, Janaki Manoja Vinnakota, Tobias Wertheimer, Philipp Saalfrank, Marie Follo, Florian Ingelfinger, and Robert Zeiser

Suppl. Fig. 1

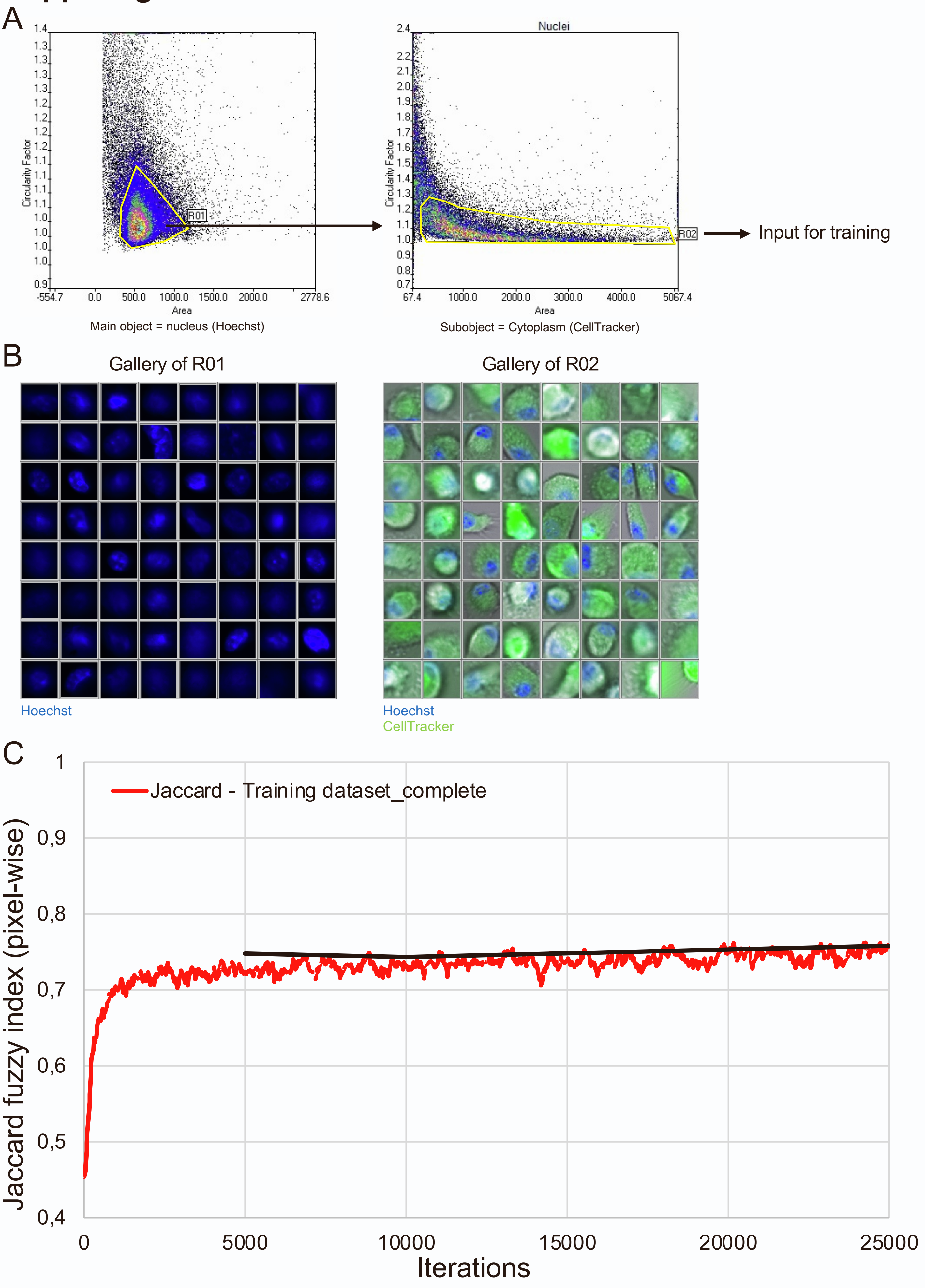
A
Circularity Factor
Area
-554.7
0.0
500.0
1000.0
1500.0
2000.0
2778.6
R01
Main object = nucleus (Hoechst)
Nuclei
Circularity Factor
Area
67.4
1000.0
2000.0
3000.0
4000.0
5067.4
R02
Subobject = Cytoplasm (CellTracker)
Input for training
B
Gallery of R01
Gallery of R02
Hoechst
Hoechst
CellTracker
C
Jaccard - Training dataset_complete
Jaccard fuzzy index (pixel-wise)
1
0,9
0,8
0,7
0,6
0,5
0,4
0
5000
10000
15000
20000
25000
Iterations

Suppl. Fig. 2

A

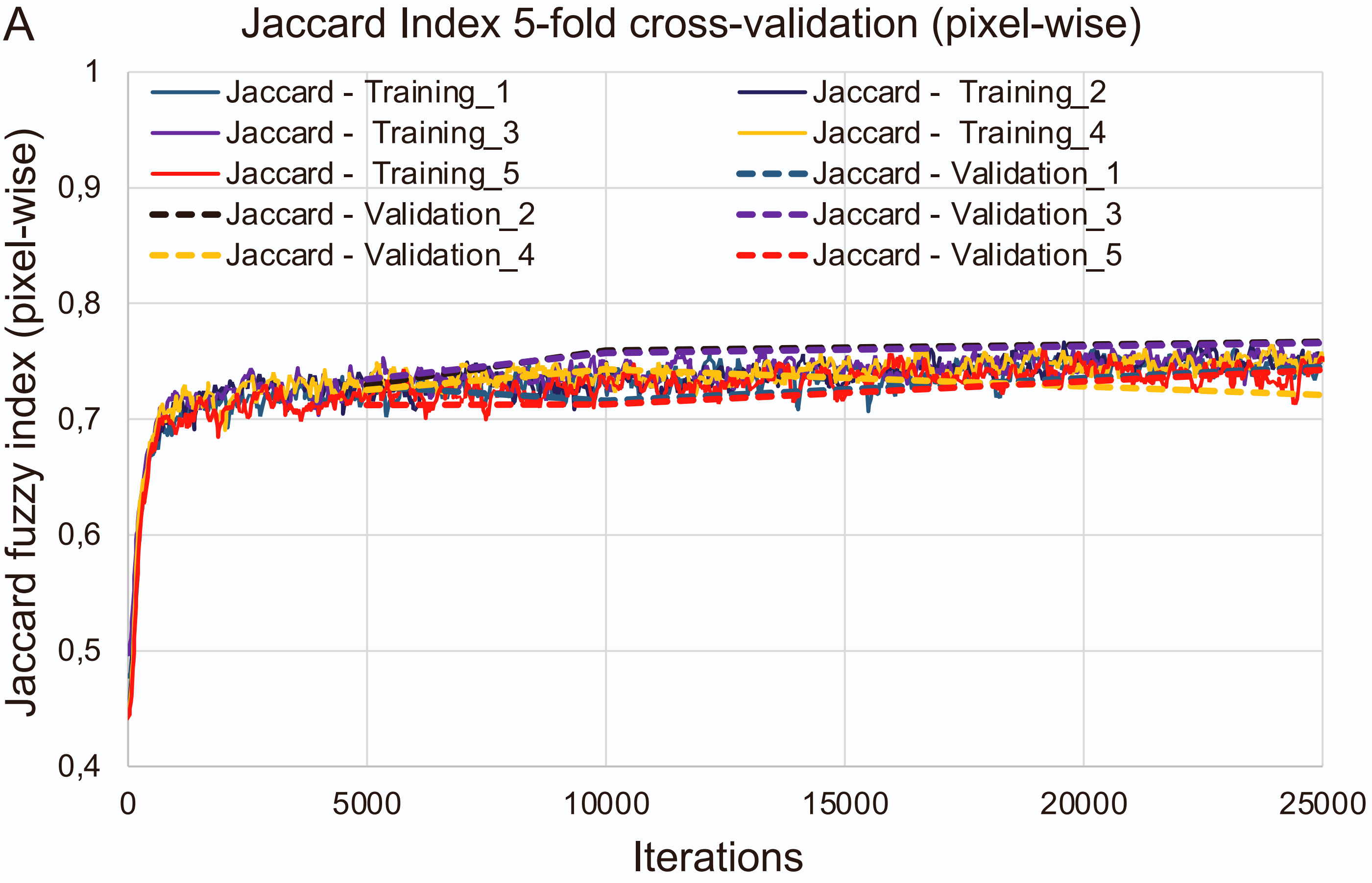

B Pooled performance metrics 5-fold cross-validation (cell-wise)

Metric	Mean	SD	SEM
**AUC**	*0.94616*	*0.01798*	*0.00804*
**Accuracy**			
t = 0	*0.99368*	*0.00332*	*0.00148*
t = 3.5 h	*0.99382*	*0.00269*	*0.00120*
**Precision**			
t = 0	*0.99509*	*0.00292*	*0.00131*
t = 3.5 h	*0.99219*	*0.00488*	*0.00218*
**Recall**			
t = 0	*0.99226*	*0.00642*	*0.00287*
t = 3.5 h	*0.99550*	*0.00199*	*0.00089*
**F1-Score**			
t = 0	*0.99366*	*0.00335*	*0.00149*
t = 3.5 h	*0.99383*	*0.00268*	*0.00119*
**Sensitivity**			
t = 0	*0.99509*	*0.00292*	*0.00131*
t = 3.5 h	*0.99218*	*0.00488*	*0.00218*

Suppl. Fig. 3

A AIstain probability map

Corresponding segmented objects

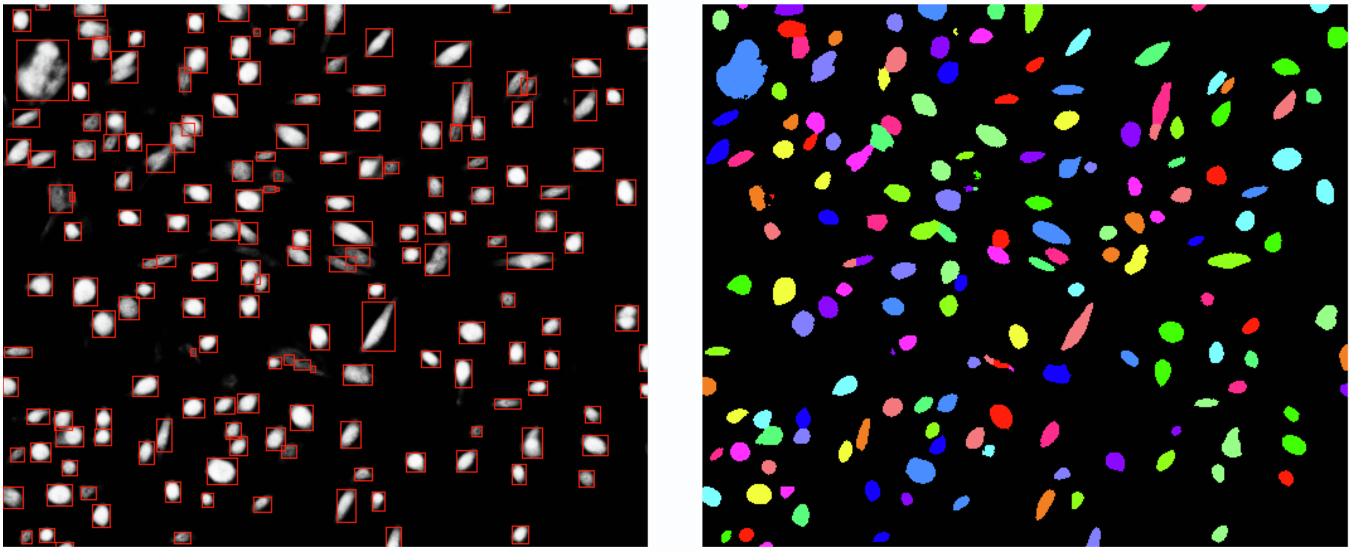

Suppl. Fig. 4

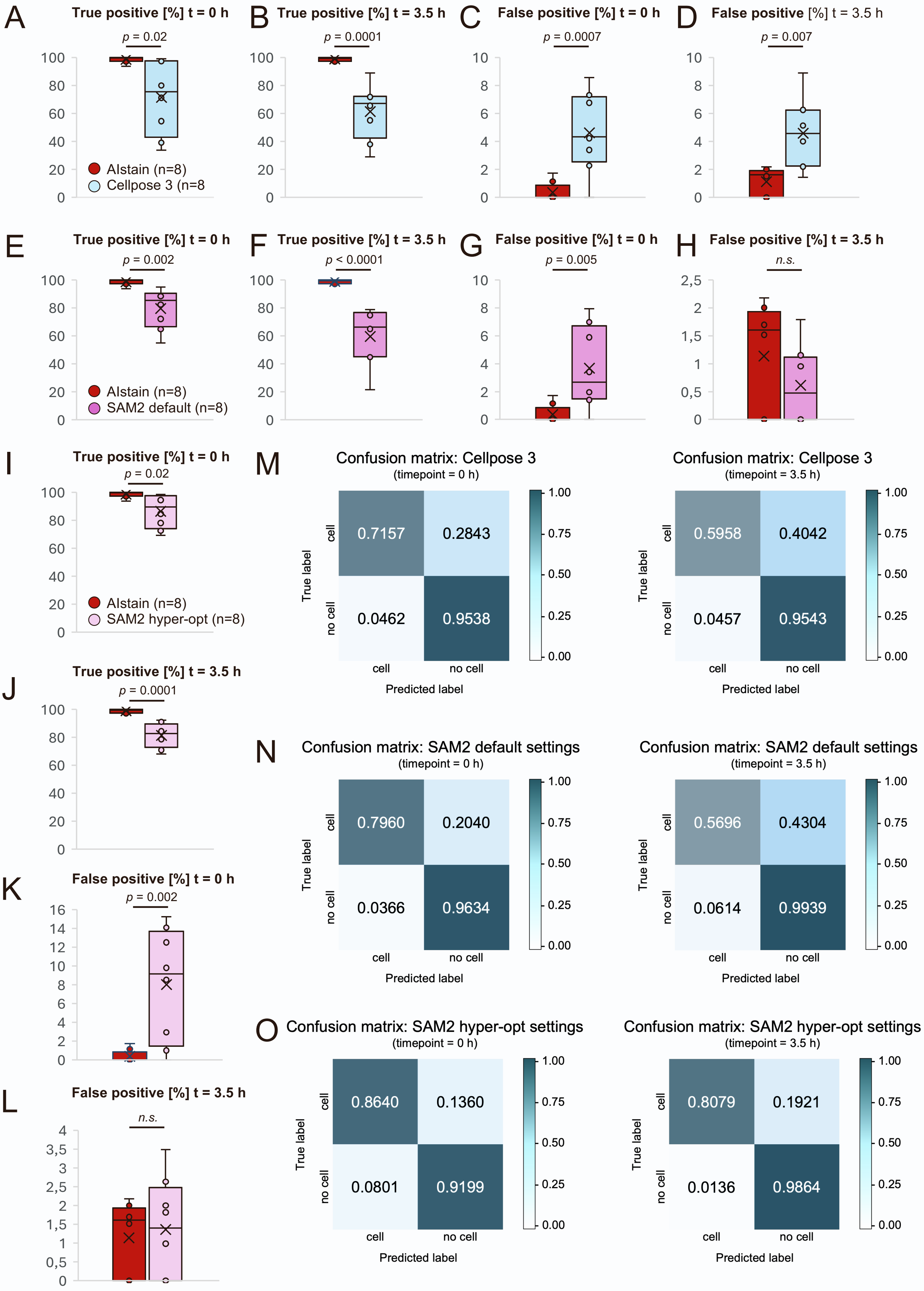

A
True positive [%] t = 0 h
p = 0.02
Alstain (n=8)
Cellpose 3 (n=8
B
True positive [%] t = 3.5 h
p = 0.0001
C
False positive [%] t = 0 h
p = 0.0007
D
False positive [%] t = 3.5 h
p = 0.007
E
True positive [%] t = 0 h
p = 0.002
Alstain (n=8)
SAM2 default (n=8)
F
True positive [%] t = 3.5 h
p < 0.0001
G
False positive [%] t = 0 h
p = 0.005
H
False positive [%] t = 3.5 h
n.s.
I
True positive [%] t = 0 h
p = 0.02
Alstain (n=8)
SAM2 hyper-opt (n=8)
J
True positive [%] t = 3.5 h
p = 0.0001
K
False positive [%] t = 0 h
p = 0.002
L
False positive [%] t = 3.5 h
n.s.
M
Confusion matrix: Cellpose 3
(timepoint = 0 h)
0.7157
0.2843
0.0462
0.9538
Confusion matrix: Cellpose 3
(timepoint = 3.5 h)
0.5958
0.4042
0.0457
0.9543
N
Confusion matrix: SAM2 default settings
(timepoint = 0 h)
0.7960
0.2040
0.0366
0.9634
Confusion matrix: SAM2 default settings
(timepoint = 3.5 h)
0.5696
0.4304
0.0614
0.9939
O
Confusion matrix: SAM2 hyper-opt settings
(timepoint = 0 h)
0.8640
0.1360
0.0801
0.9199
Confusion matrix: SAM2 hyper-opt settings
(timepoint = 3.5 h)
0.8079
0.1921
0.0136
0.9864
True label
cell
no cell
Predicted label

Suppl. Fig. 5

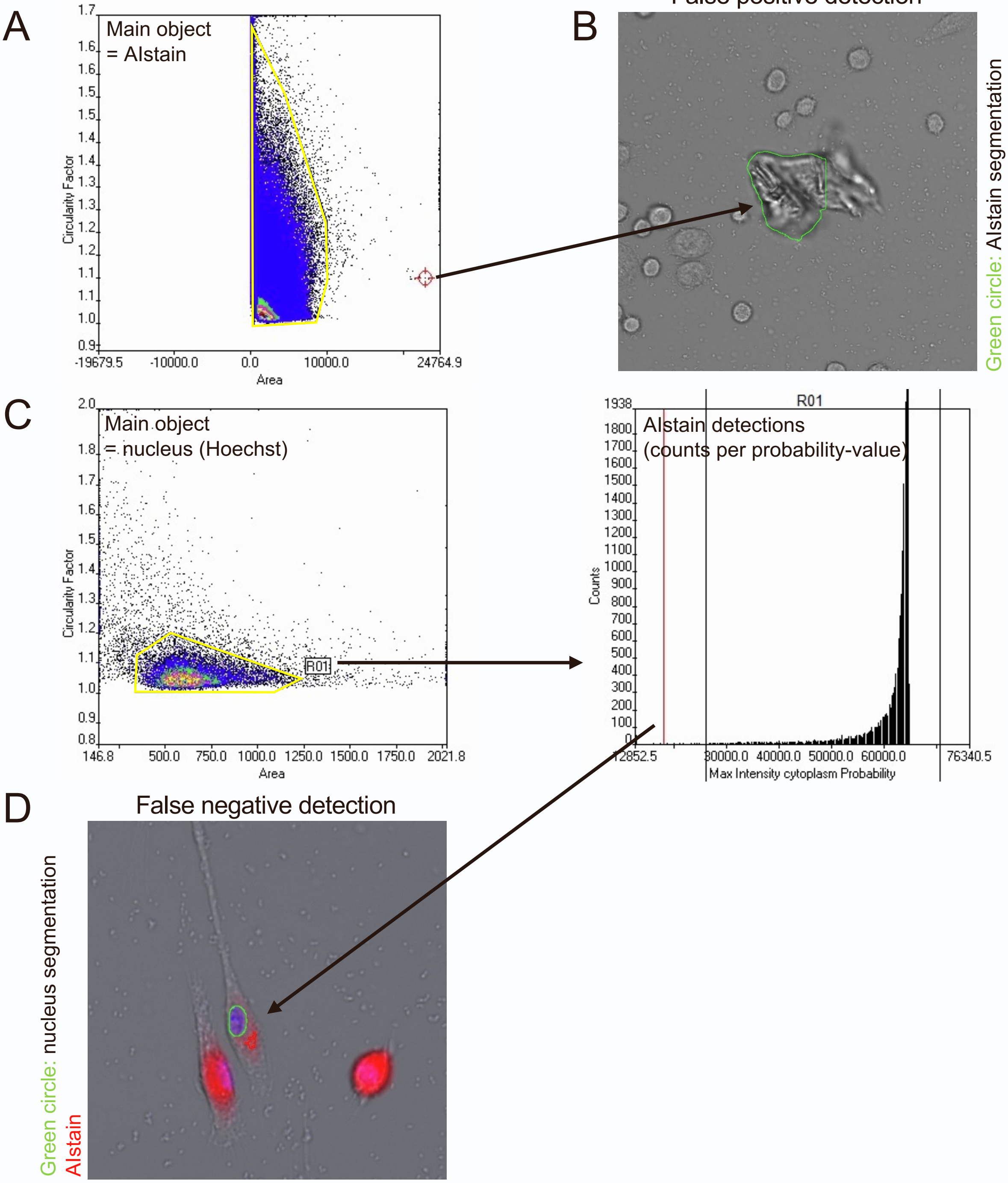

A
Main object
= Alstain
Circularity Factor
Area
-19679.5
-10000.0
0.0
10000.0
24764.9
B
False positive detection
Green circle: Alstain segmentation
C
Main object
= nucleus (Hoechst)
Circularity Factor
Area
146.8
500.0
750.0
1000.0
1250.0
1500.0
1750.0
2021.8
R01
Alstain detections
(counts per probability-value)
Counts
Max Intensity cytoplasm Probability
12852.5
30000.0
40000.0
50000.0
60000.0
76340.5
1938
D
False negative detection
Green circle: nucleus segmentation
Alstain

Supplementary figure legends:

Suppl. Fig. 1: Gating strategy in the Olympus ScanR software and representative training dynamics of AIstain, related to 'Neural network training' and 'Gating strategy' in STAR Methods.

(A) Dot plots showing the gating strategy on main object (nuclei segmentation, left) and on the subobject (CellTracker segmentation, right). Gate R01 contains true nuclei. Gate R02 contains all cells stained with CellTracker with a nucleus and physiological, microglia-like cell shape.

(B) Representative images of the gallery of gate R01 showing the nuclei (left) and R02 part of the cells around the nuclei (right). The images of the gallery were selected randomly by the Olympus ScanR software and are representative of the population inside the gate.

(C) (A) Line diagramm showing the Jaccard fuzzy index (similarity) during the training (red) and validation (black) of AIstain.

Suppl. Fig. 2: Training dynamics and model evaluation using 5-fold cross-validation, related to 'Neural network training' in STAR Methods.

(A) Line diagramm showing the Jaccard fuzzy index (similarity) during the training (solid) and validation (dashed) of the 5-fold cross-validation (pixel-wise).

(B) Table showing the pooled performance metrics of the 5-fold cross-validation (cell-wise).

Suppl. Fig. 3: AIstain probability map and segmentation, related to 'Phagocytosis Assay' in STAR Methods.

(A) Representative images of the AIstain output probability map (left) and the corresponding threshold-based segmented objects (right). Different colors indicating individual objects, each assigned with a unique object-ID.

Suppl. Fig. 4: Comparison of AIstain with Cellpose 3 and SAM2 and confusion matrices of Cellpose 3 and SAM2-based segmentation of primary microglia, related to Figure 1 C-F.

(A-L) Box plots showing percentage of true positive detection at timepoint 0h (A, E, I) of AIstain (n=8) and Cellpose 3 (n=8) (A), SAM2 default settings (n=8) (E), SAM2 hyper-opt settings (n=8) (I), at timepoint 3.5h (B, F, J) of AIstain (n=8) and Cellpose 3 (n=8) (B), SAM2 default settings (n=8) (F), SAM2 hyper-opt settings (n=8) (J), false positive detection at timepoint 0h (C, G, K) of AIstain (n=8) and Cellpose 3 (n=8) (C), SAM2 default settings (n=8) (G) SAM2 hyper-opt settings (n=8) (K) and at timepoint 3.5h (D, H, L) of AIstain (n=8) and Cellpose 3 (n=8) (D), SAM2 default settings (n=8) (H), SAM2 hyper-opt settings (n=8) (L). P-values calculated using unpaired t-test. n.s. = not significant.

(M-O) Confusion matrices showing cell segmentation of Cellpose 3 (M), SAM2 using default settings (N) and SAM2 using hyper-opt settings (O) at timepoint t = 0 h (left) and timepoint = 3.5 h (right).

Suppl. Fig. 5: Re-gating for internal control in the Olympus ScanR software, related to 'Definition of ground truth' in STAR Methods.

(A) Dot plot showing the gating strategy on the AIstain detection to identify false positive detections. All dots outside the gate are false positive detections. This example was chosen due to high numbers of false positive detections and is not representative of the AI-performance. The red cross-hair indicates a false positive detection.

(B) Representative image showing the false positive detection by AIstain (arrow and green circle).

(C) Dot plot and histogram showing the gating strategy on the AIstain detection to identify false negative detections. The left dot plot shows gating on nuclei segmentation using Hoechst. The histogram on the right side shows the distribution of the AIstain probability of objects with a segmented nucleus. The red line indicates a false negative detection.

(D) Representative image showing the false negative detection (arrow) by AIstain (red). Green circle indicates segmented nucleus.

Methods S1: Cellpose 3 Script, related to 'Cellpose 3' in STAR Methods

```
/**
 * Cellpose Detection Template script
 * @author Olivier Burri
 *
 * This script is a template to detect objects using a Cellpose model from within QuPath.
 * After defining the builder, it will:
 * 1. Find all selected annotations in the current open ImageEntry
 * 2. Export the selected annotations to a temp folder that can be specified with tempDirectory()
 * 3. Run the cellpose detction using the defined model name or path
 * 4. Reimport the mask images into QuPath and create the desired objects with the selected statistics
 *
 * NOTE: that this template does not contain all options, but should help get you started
 * See all options in https://biop.github.io/qupath-extension-cellpose/qupath/ext/biop/cellpose/CellposeBuilder.html
 * and in https://cellpose.readthedocs.io/en/latest/command.html
 *
 * NOTE 2: You should change pathObjects get all annotations if you want to run for the project. By default this script
 * will only run on the selected annotations.
 */
```

```
// Specify the model name (cyto, nuclei, cyto2, ... or a path to your custom model as a string)
// Other models for Cellpose https://cellpose.readthedocs.io/en/latest/models.html
// And for Omnipose: https://omnipose.readthedocs.io/models.html
def pathModel = 'cyto3'
def cellpose = Cellpose2D.builder( pathModel )
        .pixelSize( 1 )              // Resolution for detection in um
        .channels( 'Channel 1' )              // Select detection channel(s)
//      .tempDirectory( new File( '/tmp' ) ) // Temporary directory to export images to. defaults to 'cellpose-temp' inside the QuPath Project
//       .preprocess( ImageOps.Filters.median( 1 ) )              // List of preprocessing ImageOps to run on the images before exporting them
//              .normalizePercentilesGlobal( 0.1, 99.8, 10 ) // Convenience global percentile normalization. arguments are percentileMin, percentileMax, dowsample.
//      .tileSize( 1024 )              // If your GPU can take it, make larger tiles to process fewer of them. Useful for Omnipose
//      .cellposeChannels( 1,2 )        // Overwrites the logic of this plugin with these two values. These will be sent directly to --chan and --chan2
//      .cellprobThreshold( 0.0 )        // Threshold for the mask detection, defaults to 0.0
//      .flowThreshold( 0.4 )            // Threshold for the flows, defaults to 0.4
        .diameter( 50 )               // Median object diameter. Set to 0.0 for the `bact_omni` model or for automatic computation
//      .useOmnipose()               // Use omnipose instead
//      .addParameter( "cluster" )      // Any parameter from cellpose or omnipose not available in the builder.
```

```
//        .addParameter( "save_flows" )       // Any parameter from cellpose or omnipose not available in the builder.
//        .addParameter( "anisotropy", "3" ) // Any parameter from cellpose or omnipose not available in the builder.
//      .cellExpansion( 5.0 )            // Approximate cells based upon nucleus expansion
//      .cellConstrainScale( 1.5 )        // Constrain cell expansion using nucleus size
//      .classify( "My Detections" )      // PathClass to give newly created objects
//      .measureShape()                // Add shape measurements
//      .measureIntensity()            // Add cell measurements (in all compartments)
//        .createAnnotations()             // Make annotations instead of detections. This ignores cellExpansion
//      .simplify( 0 )                 // Simplification 1.6 by default, set to 0 to get the cellpose masks as precisely as possible
        .build()
// Run detection for the selected objects
def imageData = getCurrentImageData()
def pathObjects = getSelectedObjects() // To process only selected annotations, useful while testing
// def pathObjects = getAnnotationObjects() // To process all annotations. For working in batch mode
if (pathObjects.isEmpty()) {
    Dialogs.showErrorMessage( "Cellpose", "Please select a parent object!" )
    return
```

```
}

cellpose.detectObjects( imageData, pathObjects )

// You could do some post-processing here, e.g. to remove objects that are too small, but it is
usually better to
// do this in a separate script so you can see the results before deleting anything.

println 'Cellpose detection script done'

import qupath.ext.biop.cellpose.Cellpose2D
```

Methods S2: SAM 2 settings, related to 'SAM2' in STAR Methods.

Default settings:

- SAM model: sam2_l (large)
- SAM weights: default
- Auto mask
- Points per side 64
- Points per batch: 64
- Pred IoU thresh: 0.88
- Stability score thresh: 0.95
- Stability score offset: 1
- Box NMS thresh: 0.2
- Crop N layers: 0
- Crop NMS thresh: 0.7
- Crop overlap ratio: 0.34
- Crop N points downscale factor: 1
- Min mask region area: 0

Hyper-opt settings:

- SAM model: sam2_l (large)
- SAM weights: default
- Auto mask
- Points per side 64
- Points per batch: 64
- Pred IoU thresh: 0.7

- Stability score thresh: 0.95
- Stability score offset: 1
- Box NMS thresh: 0.2
- Crop N layers: 0
- Crop NMS thresh: 0.7
- Crop overlap ratio: 0.34
- Crop N points downscale factor: 1
- Min mask region area: 0
